# Novel implant-free loop Tenodesis vs. simple Tenotomy of the long biceps tendon - a biomechanical investigation

**DOI:** 10.1186/s12891-019-2919-z

**Published:** 2019-11-09

**Authors:** Maximilian Kerschbaum, Andreas Voss, Christian Pfeifer, Isabella Weiss, Agnes Mayr, Volker Alt, Stefan Greiner, Stephan Grechenig

**Affiliations:** 1 Clinic of Trauma Surgery, University Medical Center Regensburg, Franz-Josef-Strauss-Allee 11, 93053 Regensburg, Germany; 2 Sporthopaedicum Regensburg, Hildegard von Bingen Strasse 1, 93053 Regensburg, Germany

**Keywords:** Loop Tenodesis, Biceps tenotomy, Biomechanical evaluation, LHB, Popeye deformity, Long head of biceps

## Abstract

**Background:**

Simple tenotomy and anchor tenodesis are commonly used in treatment of long biceps tendon (LHB) pathologies. The tenotomy can result in biceps distalization or cosmetic deformities. A novel loop Tenodesis Technique (LTD) could prevent a distalization of the biceps muscle without the risk of implant associated complications. The purpose of this study was to investigate the biomechanical aspects of the novel LTD compared to a standard tenotomy of the LHB. It has been hypothesized that the novel technique will show biomechanical superiority in terms of resistance and distalization.

**Methods:**

Seven paired adult human cadaveric shoulder joints were assigned to one of the two study groups: Loop tenodesis (LTD); simple tenotomy (STT). In both groups load-to-failure testing was performed. The load-displacement curve was used to determine the maximum load (N), the degree of distalization of the LHB (mm) and the stiffness (N/mm). Additionally, the mode of failure was registered.

**Results:**

The LTD group achieved a significantly higher ultimate load to failure (LTD: 50.5 ± 12.5 N vs. STT: 6.6 ± 3.9 N; *p* = 0.001). Significantly less distalization of the tendon could be detected for the LTD group (LTD: 8 ± 2.3 mm vs. STT: 22.4 ± 2.4 mm; p = 0.001). Stiffness was 7.4 ± 3.9 N/mm for the LTD group and 0.23 ± 0.16 N/mm for the STT group (p = 0.001). In all specimens of the LTD group a tendon rupture was found as mode of failure, while the STT group failed because of pulling out the LHB through the bicipital groove.

**Conclusion:**

The novel loop Tenodesis Technique shows biomechanically higher stability as well as less distalization compared to a simple tenotomy of the long biceps tendon.

## Background

Anterior shoulder pain is often caused by pathologies of the long biceps tendon [1, 2]. In order to treat such pathologies many different arthroscopic and open techniques have been described. In addition to a simple tenotomy of the tendon, various tenodesis techniques were published [3, 4]. A well-known problem of simple LHB tenotomy is distalization of the tendon, which can lead to muscle cramps as well as cosmetic deformities of the upper arm [5, 6]. Therefore, many studies recommend tenodesis of the LHB in contrast to a simple tenotomy to prevent a distalization of the tendon in order to reduce the complications mentioned above [7]. Various clinical studies have shown a significantly better result, especially in cosmetic concerns [8].

In particular, techniques, with tendon-to-bone fixation by using a suture anchor or tenodesis screw, were found to be biomechanically and clinically superior to soft-tissue fixation techniques [9]. However, there are some complications that can occur when performing an anchor tenodesis of the long biceps tendon. Persistent pain has often been observed in the anchor insertion area. In addition, cases of fractures and infections caused by the inserted foreign materials are described [7, 8, 10–12].

Thus, the purpose of the present study is to biomechanically evaluate a new, implant-free tenodesis technique, called loop Tenodesis (LTD) compared to a standard tenotomy (STT). It has been hypothesized that the novel technique will show biomechanical superiority in terms of tear resistance and distalization.

## Methods

### Specimens

**Fig. 1:**
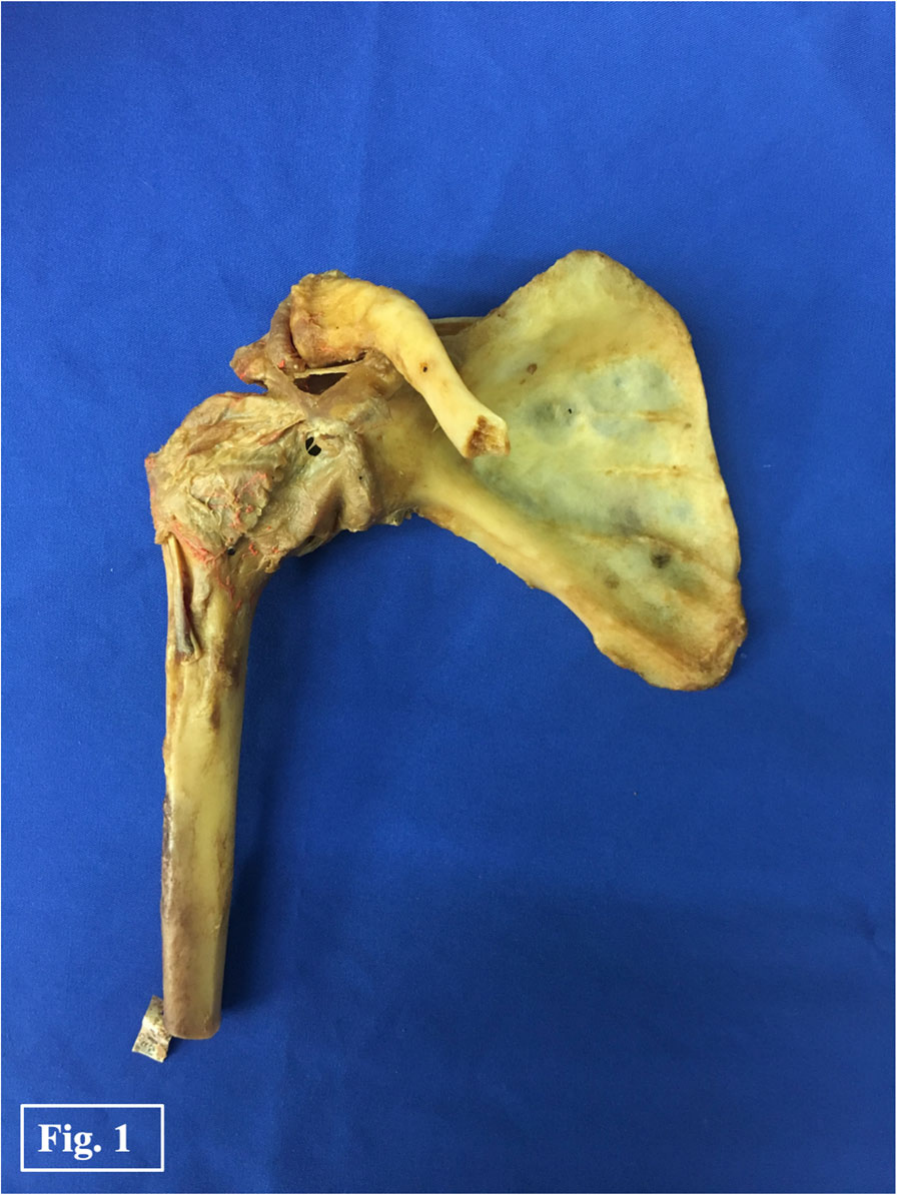
Dissected specimens: Human cadaveric shoulder with preserved joint capsule and biceps pulley

A total of 7 paired human adult cadaveric shoulder joints preserved by Thiel’s method [13] were used in the current study. The embalming method preserves colour, consistency and biomechanical properties of tissues. Cadaveric specimens were donated to the university anatomy program. Donors gave their informed consent within the donation of anatomical gift statement during their lifetime. None of the shoulders showed signs of previous injuries, abnormalities or diseases. The specimens were dissected and stripped of surrounding muscles. Ligaments, joint capsule and the pulley complex (superior glenohumeral ligament (SGHL), the coracohumeral ligament (CHL), and fibers blending in from the subscapularis and supraspinatus tendons) [14] were preserved (Fig. 1). The long head of the biceps (LHB) remained unscathed.

One shoulder of each pair (left or right) was randomly assigned to the LHB loop tenodesis group (LTD group), whereas the contralateral shoulder was assigned to the LHB tenotomy group (STT group). As a result, two groups with seven specimens each were assigned to achieve an intra-individual comparison resulting in a matched pair study design. Surgery was performed in a fashion simulating the arthroscopic technique.

### Tenotomy of the LHB

The LHB was tenotomized close to its origin. The tendon was released, followed by a spontaneous retraction with subsequent autotenodesis in the sulcus [15, 16].

### Loop tenodesis of the LHB

The loop tenodesis procedure was performed as recently described by Kerschbaum et al. 2019 [17].

**Fig. 2:**
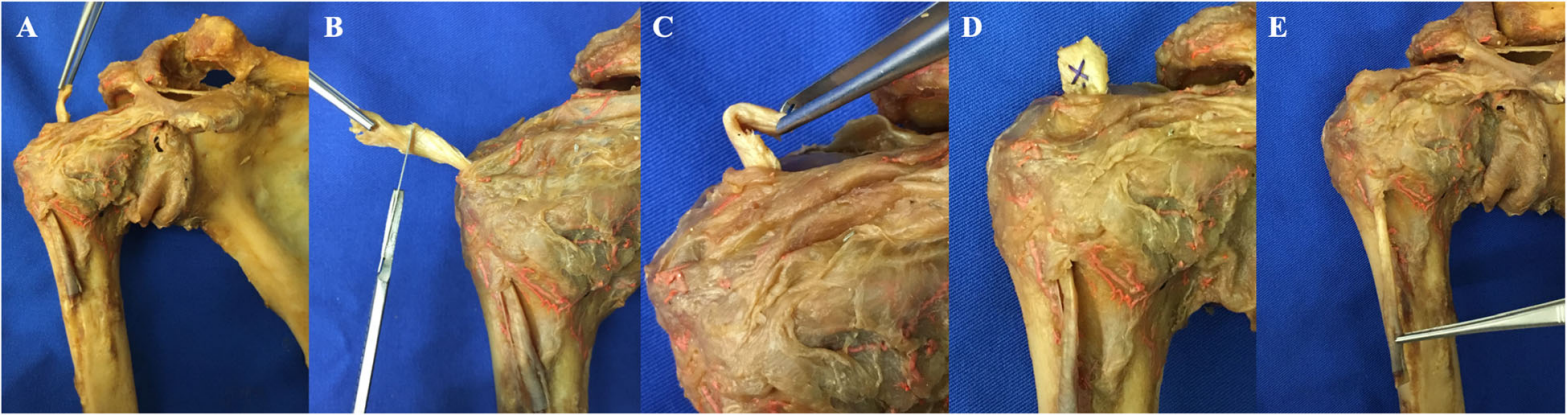
Novel loop tenodesis: **a**) Long biceps tendon (LHB) pulled extraarticularly after tenotomy close to its base; **b**) Resection of the proximal part of the LHB; **c**) Tendon loop; **d**) Suturing the tendon loop; **e**) Autotenodesis of the LHB

After tenotomy of the LHB close to its origin, the tendon was pulled extraarticularly through an anterolateral portal with a clamp (Fig. 2a). The proximal part (1 cm) was resected (Fig. 2b). A tendon loop was created by folding the tip of the tendon (Fig. 2c) and sutured with Vicryl 3.0 (Johnson & Johnson Medical GmbH, Ethicon, Norderstedt, Germany) (Fig. 2d). The tendon was then released, followed by a spontaneous retraction with subsequent autotenodesis in the sulcus (Fig. 2e) [15, 16].

### Biomechanical testing

**Fig. 3:**
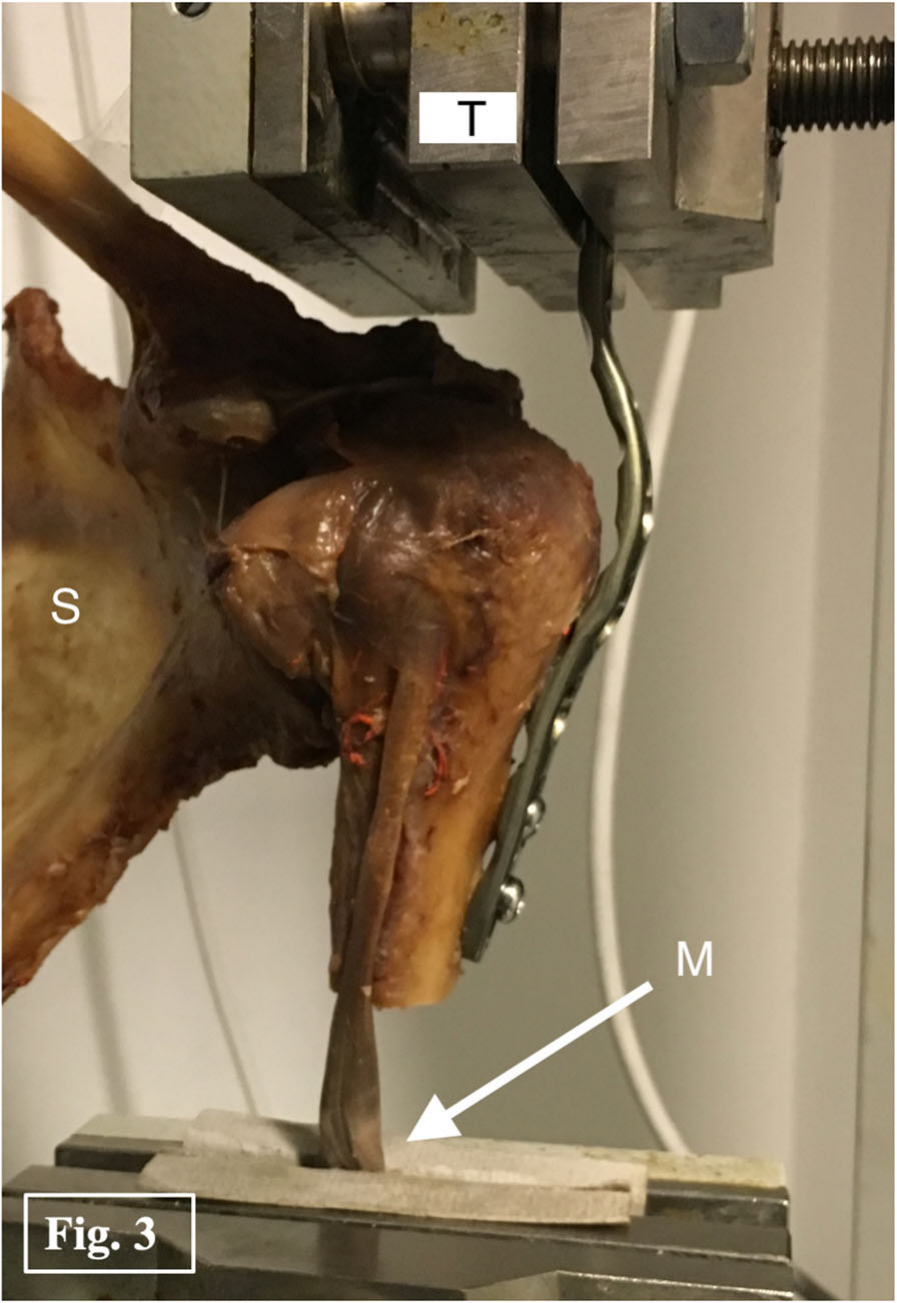
Biomechanical test setup: In line force application (S = scapula, T = testing machine, M = musculotendinous junction).

For biomechanical testing, a special bent plate was fixed to the humerus and attached to the material testing machine by a clamp. The long biceps tendon was also attached to the testing machine at the musculotendinous junction with a special clamp. Force application was performed in line with the tendon and the humeral shaft (Fig. 3). A preload of 1 N was applied for 5 s to pre-tension the LHB. A load to failure protocol was used with a displacement rate of 1 mm/s. In order to guarantee constant tissue properties, all samples were kept moist at room temperature during biomechanical testing. Ultimate load (N), failure displacement (mm) and failure mode were recorded. To get information about the stiffness of the construct (N/mm) the slope of the increasing part of the load-displacement curve was determined as decribed by Lopez-Vidriero et al. [18].

### Statistics

Statistical analysis was carried out using SPSS software (SPSS Inc., Chicago, Illinois). Normal distribution assumption was not verified thus the mean differences between the biomechanical variables of the two techniques were evaluated using the Mann–Whitney test. All data were given in mean ± standard deviations. *P*-values <0.05 were considered significant.

## Results

**Fig. 4:**
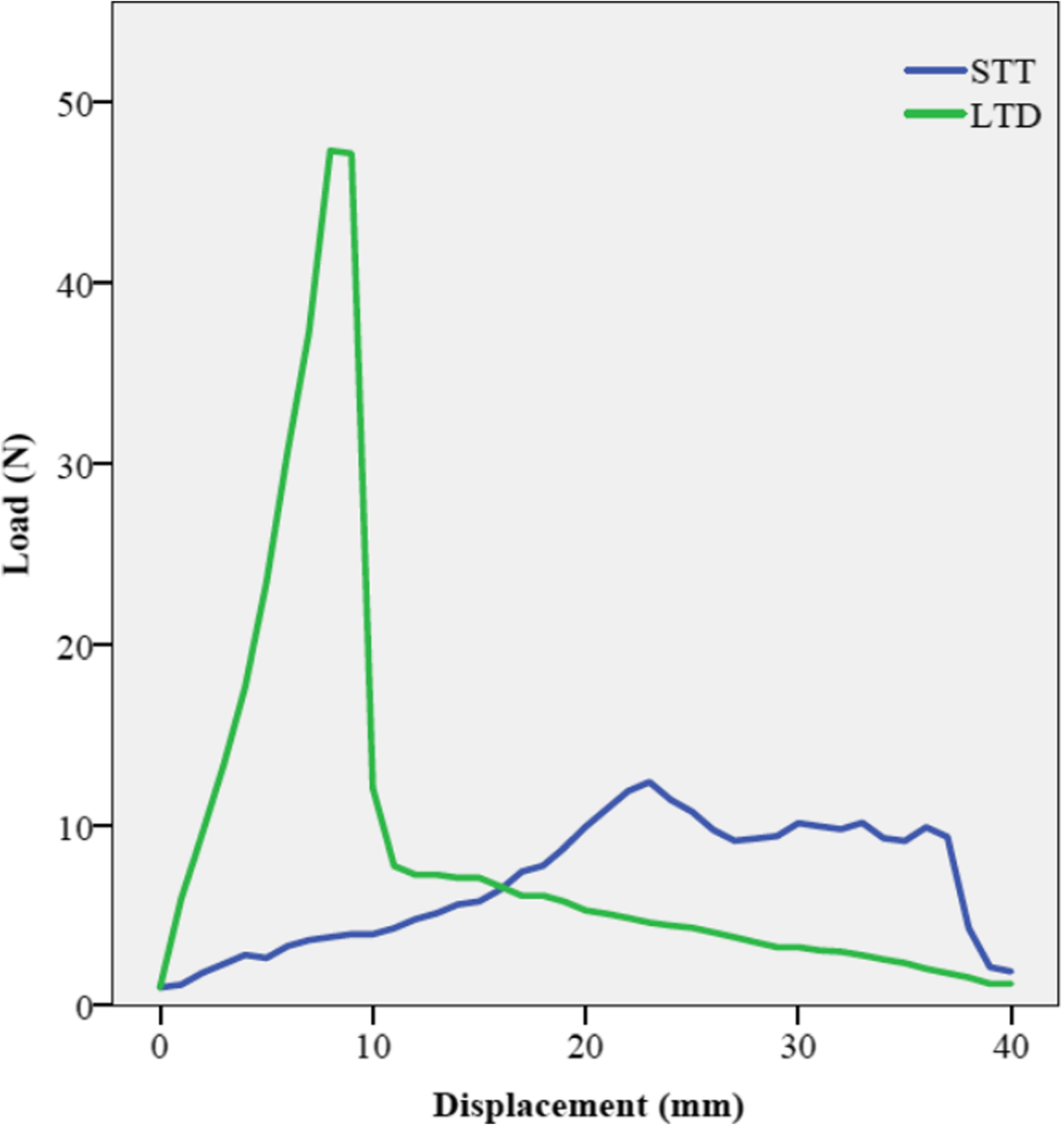
Load-displacement curve (illustrated on one specimen): STT: Simple tenotomy; LTD: Loop tenodesis

There was a rapid increase in force until the ultimate load to failure was reached in the LTD group. After the failure occurred, there was a rapid decrease in strength (Fig. 4).

The load-displacement curve of the STT group showed an initial increase of load followed by a plateau phase, which quickly dropped after the LHB was completely slipped out of the bicipital groove and the pulley complex (Fig. 4).

**Fig. 5:**
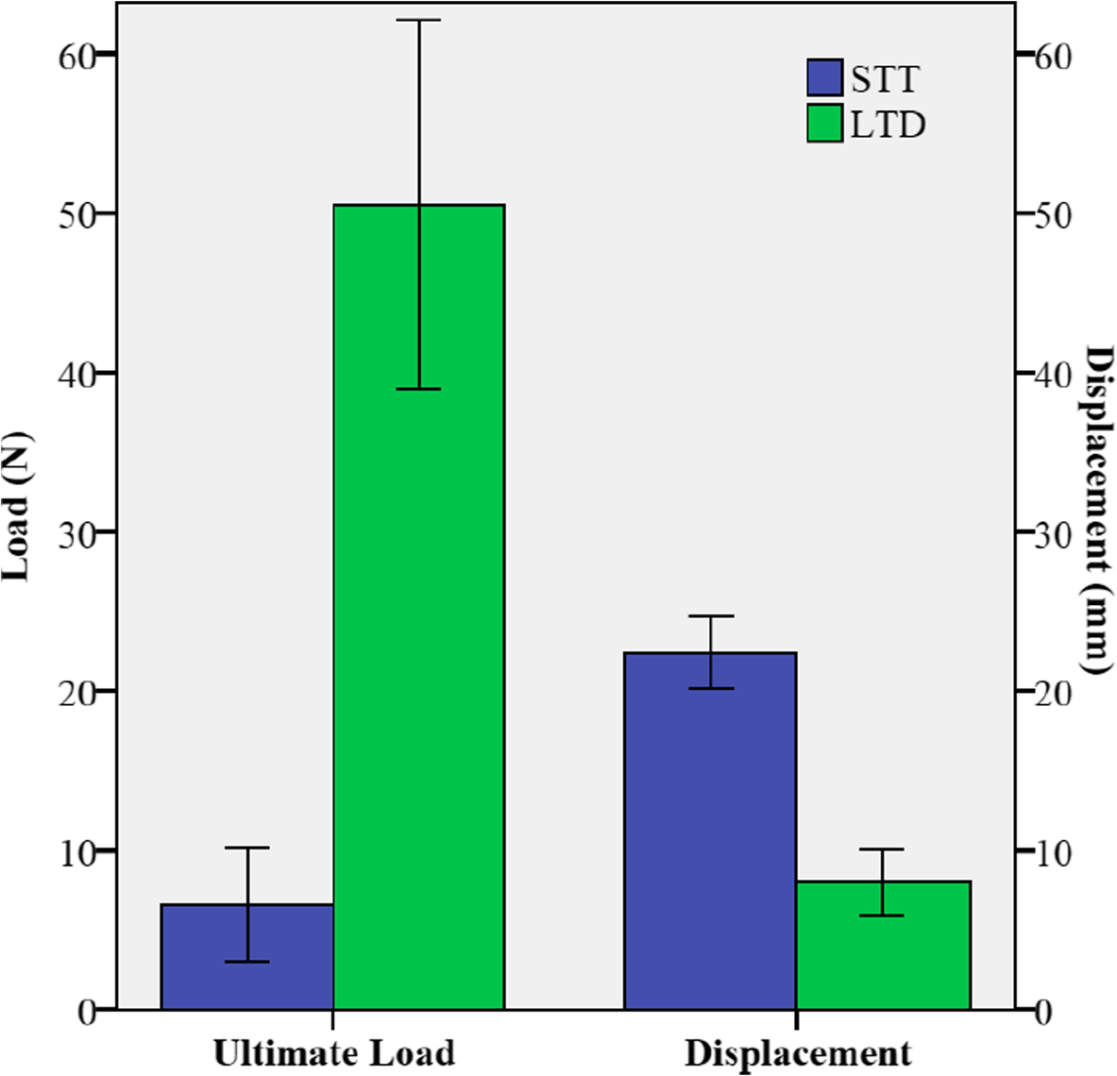
Ultimate load / Displacement of loop tenodesis and simple tenotomy

The ultimate load of the LTD group was significantly higher compared to the STT group (LTD: 50.5 ± 12.5 N vs. STT: 6.6 ± 3.9 N; *p* = 0.001). At this point the displacement of the tendon was significantly lower in the LTD group compared to the STT group (LTD: 8 ± 2.3 mm vs. STT: 22.4 ± 2.4 mm; p = 0.001) (Fig. 5).

The calculated stiffness was 7.4 ± 3.9 N/mm for the LTD group and 0.23 ± 0.16 N/mm for the STT group (*p* = 0.001). In all specimens of the LTD group a complete tendon rupture distal of the loop was found, while the STT group failed because of a complete pull out of the LHB through the bicipital groove.

## Discussion

The most important findings of this study showed a biomechanical superiority in terms of distalization and load to failure for the novel loop tenodesis compared to a standard tenotomy. Hence the hypothesis could be verified.

Both, tenotomy and tenodesis are established methods for the treatment of long biceps tendon pathologies. To what extent tenodesis of the LHB is able to prevent complications due to a distalization of the tendon compared to a simple tenotomy has been discussed repeatedly [6, 7, 19]. Some studies have shown that LHB tenodesis is superior compared to simple tenotomy, especially concerning the cosmetic result, with patients treated by tenodesis exhibiting significantly less upper arm deformities [7, 8]. In particular, bony fixation techniques were able to demonstrate superior results compared to soft-tissue tenodesis procedures or a simple tenotomy [6, 9].

Nevertheless, some complications of these tenodesis procedures are described. Next to fractures, implant dislocation and nerve injuries, persistent anterior shoulder pain at the anchor insertion site are known complications [7, 8, 10–12].

In contrast, other studies could not prove significant advantages of tenodesis procedures over a simple tenotomy. Osbahr et al. did not find any significant differences between both procedures regarding the cosmetic result [15]. An own investigation could show that a complete slippage of the tenotomized biceps tendon through the bicipital groove only occurs in very few patients [6]. These results suggest that the long biceps tendon has the ability to fix itself in the bicipital groove. In 2012 Kim et al. described this phenomenon as autotenodesis of the long biceps tendon and could show that adhesions in the bicipital groove can prevent distalization [16]. Also, the “Vinculae”, described by Johnson et al. in 1992, which cross-connect the long biceps tendon and the bicipital groove support the thesis of the possibility of an autotenodesis of the long biceps tendon [20]. The novel loop tenodesis is based on the autotenodesis phenomenon. By enlarging the diameter of the tendon at the entrance to the bicipital groove, the self-locking mechanism of the long biceps tendon is supported, which prevents the LHB from distalization.

In order to evaluate the new technique biomechanically, human cadaveric shoulders were used.

While the majority of biomechanical investigations on related topics dissected the surrounding soft tissues and did not preserve the pulley complex, which is relevant for in vivo stability, both the capsule apparatus and the pulley complex were protected in the present study [18, 21–26]. In this way, structures influencing biomechanics in vivo could be preserved [14]. By using a human cadaveric model in matched paired design, the influence of anatomical variability in specimen’s parameters was considerably reduced, making it possible to compare the intrinsic biomechanical performance of the groups.

All samples of the tenotomy group showed a slippage of the tendon out of the bicipital groove as failure mode. A closer look at the load-displacement curve of the tenotomy group reveals, that a plateau phase followed the ultimate load, during which there was an increasing distalization of the tendon without any relevant increase in load. However, the level of autotenodesis capability can also be shown, because the load curve did not drop to zero level during the slippage of the tendon.

In contrast to that, the loop tenodesis group showed a rapid increase in force till tear of the tendon occurred. The rapid increase in load is indicative for tendon blocking at the entrance to the sulcus bicipitalis and indicates a high primary stability of this technique. The fact that all specimens failed due to a tear and not due to a slippage of the tendon supports the hypothesis of a high primary biomechanical stability of the present technique. Another key finding is that the new technique, compared to a tenotomy, achieves the ultimate load at a much earlier stage.

Clinical data showed that even though the LHB does not completely slip down after tenotomy, in 69% an objective upper arm deformity occurs [6].

This result supports the hypothesis of autotenodesis capability of the long biceps tendon, but with a disturbed length-tension-relationship. Tightening of the tendon further proximal and therefore with a more physiological length-tension-relationship, as biomechanically shown in the novel loop tenodesis, could clinically reduce the presence of upper arm deformities. This fact is also evident in stiffness of the constructs, whereby the loop tenodesis achieved significantly higher values. Nevertheless, the elasticity of the tendon, which has an influence on stiffness, must also be taken into account. In order to reduce differences due to different elasticities in tested tendons, all LHBs were clamped at musculotendinous transition zone. Whether the LHB loop can cause complaints at the bicipital groove must be clarified in clinical studies.

However, the limitations of this study must be discussed in detail. This is a study on cadaveric specimens, which cannot simulate healing processes or the influence of post-treatment strategies. In addition, the embalming process can have influence on the biomechanical properties of the specimens. A further limitation of investigations on human cadaveric specimens is that the biomechanical influence of a tendon disease, which usually indicates tenotomy or tenodesis of the LHB, cannot be taken into account.

Despite the limitations of this study, biomechanical superiority (ultimate load and stiffness) of the novel, implant-less loop tenodesis technique compared to a simple tenotomy could be demonstrated.

Further studies are necessary to evaluate this technique in a clinical setting.

## Conclusion

Compared to a simple tenotomy of the LHB, the novel, implant-free loop tenodesis technique achieved a higher ultimate load and stiffness with less distalization in biomechanical setting.

## Data Availability

The datasets used during the current study are available from the corresponding author on reasonable request.

## Abbreviations

CHL: Coracohumeral ligament
LHB: Long head of biceps tendon
LTD: Loop tenodesis procedure
mm: Millimeter
N: Newton
SGHL: Superior glenohumeral ligament
STT: Simple tenotomy
